# High-Rate Sulfate Removal Coupled to Elemental Sulfur Production in Mining Process Waters Based on Membrane-Biofilm Technology

**DOI:** 10.3389/fbioe.2022.805712

**Published:** 2022-03-07

**Authors:** Alex Schwarz, María Gaete, Iván Nancucheo, Denys Villa-Gomez, Marcelo Aybar, Daniel Sbárbaro

**Affiliations:** ^1^ Civil Engineering Department, Universidad de Concepción, Concepción, Chile; ^2^ Facultad de Ingeniería y Tecnología, Universidad San Sebastián, Concepción, Chile; ^3^ School of Civil Engineering, The University of Queensland, Brisbane, QLD, Australia; ^4^ Electrical Engineering Department, Universidad de Concepción, Concepción, Chile

**Keywords:** membrane biofilm reactor, sulfur-oxidizing bacteria, sulfate, elemental sulfur, mine tailings, sulfate-reducing bacteria

## Abstract

It is anticipated that copper mining output will significantly increase over the next 20 years because of the more intensive use of copper in electricity-related technologies such as for transport and clean power generation, leading to a significant increase in the impacts on water resources if stricter regulations and as a result cleaner mining and processing technologies are not implemented. A key concern of discarded copper production process water is sulfate. In this study we aim to transform sulfate into sulfur in real mining process water. For that, we operate a sequential 2-step membrane biofilm reactor (MBfR) system. We coupled a hydrogenotrophic MBfR (H_2_-MBfR) for sulfate reduction to an oxidizing MBfR (O_2_-MBfR) for oxidation of sulfide to elemental sulfur. A key process improvement of the H_2_-MBfR was online pH control, which led to stable high-rate sulfate removal not limited by biomass accumulation and with H_2_ supply that was on demand. The H_2_-MBfR easily adapted to increasing sulfate loads, but the O_2_-MBfR was difficult to adjust to the varying H_2_-MBfR outputs, requiring better coupling control. The H_2_-MBfR achieved high average volumetric sulfate reduction performances of 1.7–3.74 g S/m^3^-d at 92–97% efficiencies, comparable to current high-rate technologies, but without requiring gas recycling and recompression and by minimizing the H_2_ off-gassing risk. On the other hand, the O_2_-MBfR reached average volumetric sulfur production rates of 0.7–2.66 g S/m^3^-d at efficiencies of 48–78%. The O_2_-MBfR needs further optimization by automatizing the gas feed, evaluating the controlled removal of excess biomass and S^0^ particles accumulating in the biofilm, and achieving better coupling control between both reactors. Finally, an economic/sustainability evaluation shows that MBfR technology can benefit from the green production of H_2_ and O_2_ at operating costs which compare favorably with membrane filtration, without generating residual streams, and with the recovery of valuable elemental sulfur.

## Highlights

• Sulfate conversion to S^0^ in real mining process water was achieved using MBfRs
• The H_2_-MBfR achieved high sulfate removals of 1.7–3.74 g S/m^3^-d efficiently
• The O_2_-MBfR produced S^0^ at efficiencies of 48–78%, needing further automatization
• The economic evaluation gives a competitive green H_2_ gas cost of 0.17 US$/kg SO_4_
• MBfR technology is safer because it minimizes H_2_S and H_2_ off-gassing risks

## Introduction

It is expected that the current 21 Mt copper mining output will increase by 28% over the next 20 years, as the demand for copper from clean energy technologies grows by a factor of up to 2.7 in line with the Paris Agreement goals (IEA, 2021). Copper intensity is significantly higher in electricity-related technologies for transport (24 kg/vehicle in conventional cars versus 53 kg/vehicle in electric cars) and power generation (1,100–1,150 kg/MW in natural gas-coal versus 2,800–8,000 kg/MW in solar photovoltaic-offshore wind), while expanded electricity networks will also need significant amounts of copper. Hence, the environmental impacts associated with copper mineral mining and processing are expected to rise accordingly unless environmental mining regulations become stricter and as a result, cleaner mining technologies are implemented. Particularly, the impacts of copper mining on water resources are twofold. The oxidation of residual metal sulfides in waste rock produces acid mine drainage characterized by acidic pH and high metals and sulfate concentrations (Lottermoser, 2007; Dold, 2010). Similarly, sulfated process waters are commonly discarded along with mineral tailings in unlined surface impoundments (Schwarz et al., 2020). Both effluents often contaminate surface and ground waters, and because of the large scale of some mining operations, the effects on wildlife and human health can be significant (Simate and Ndlovu, 2014).

To remove the sulfate present in mining effluents, biological treatment has been regarded as a more cost-effective option compared to chemical and physicochemical processes (Skousen et al., 2019). Biological sulfate removal is a two-step process, involving an initial reduction of sulfate to sulfide, followed by the oxidation of sulfide to elemental sulfur (S^0^). Because sulfide can be used as a ligand for sequential precipitation of metals and S^0^ has agronomic value, biological treatment can be applied for resource recovery (Kaksonen and Puhakka, 2007; Lin et al., 2018; Giordano et al., 2019; Kisser et al., 2020).

With H_2_ as the inorganic electron donor, the biological reduction reaction of sulfate to sulfide is (Muyzer and Stams, 2008):

$$4\;{\text{H}}_{2}\;+\;\text{S}{\text{O}}_{4}^{2-}+\;{\text{H}}^{+}\to \text{H}{\text{S}}^{-}\;+\;4\;{\text{H}}_{2}\text{O}\;\;\;\Delta {\text{G}}^{0\prime }\;=\;-151\text{.}90\;\text{kJ}/\text{reaction}\;\left(-19\text{.0 kJ}/{\text{e}}^{-}\right)$$ (1)

H_2_ is a good electron donor substrate for the treatment of inorganic sulfate-rich waters because it is cheap (Bijmans et al., 2011), clean and non-toxic, and can be produced on-site using green electricity (Acar and Dincer, 2018). Furthermore, H_2_ utilization efficiency can be optimized for sulfate reduction by limiting the CO_2_ feed to control methanogenesis (van Houten et al., 2009). With O_2_ as the electron acceptor, the sulfide-oxidation reactions can be summed up as follows (Madigan et al., 2019):

$$HS^{-}+H^{+}+0.5\;O_{2}\to \;{\text{S}}^{0}\;+\;H_{2}O\;\;\;\Delta G^{0\prime }\;=\;-209\text{.}4\;\text{kJ}/\text{reaction}\;\left(-104\text{.}7\text{ kJ}/{\text{e}}^{-}\right)$$ (2)

$$S^{0}+1.5\;O_{2}\;+\;H_{2}O\to SO_{4}^{2-}\;+\;2\;H^{+}\;\;\;\Delta G^{0\prime }\;=\;-587\text{.}1\;\text{kJ}/\text{reaction}\;\left(-97\text{.}9\text{ kJ}/{\text{e}}^{-}\right)$$ (3)

$$HS^{-}\;+\;2\;O_{2}\to \;SO_{4}^{2-}\;+\;{\text{H}}^{+}\;\;\;\Delta G^{0\prime }\;=\;-796\text{.}5\;\text{kJ}/\text{reaction}\;\left(-99.6\text{kJ}/{\text{e}}^{-}\right)$$ (4)

Reaction 2 of incomplete oxidation of sulfide to S^0^ is the desirable reaction as sulfide is not reoxidized to sulfate and only 25% of the O_2_ is consumed, and because S^0^ is a valuable end product. S^0^ formation is favored under O_2_ limitation (∼0.1 mg O_2_/L), and S^0^ yields >90% can be reached (Lin et al., 2018).

Dissimilatory sulfate reduction (Eq. 1) is mostly catalyzed by sulfate-reducing bacteria (SRB) belonging to the phylum *Desulfobacterota* (Waite et al., 2020). Particularly the genus *Desulfovibrio* is often dominant in sulfate-reducing reactors (Dar et al., 2007; Schwarz et al., 2020) under pH neutral conditions, though in acidic environments, species within the *Peptococcaceae* family of the *Firmicutes* phylum have been described (Sánchez-Andrea et al., 2015). Sulfide oxidation (Eqs. 2-4) used in biotechnological applications, on the other hand, commonly involves sulfur-oxidizing bacteria (SOB) of the genera *Thioalkalimicrobium*, *Thioalkalivibrio*, or *Thiobacillus* (Janssen et al., 1995; van den Bosch et al., 2007; Sorokin et al., 2013; Muyzer et al., 2013).

While sulfate removal systems have been well studied and even commercial systems exist (Kaksonen and Puhakka, 2007; Hao et al., 2014), they continue to be optimized (Mora et al., 2020). New applications are also constantly emerging including the fluidized bed membrane bioreactor (Oztemur et al., 2020), the membrane biofilm reactor (Schwarz et al., 2020), and the sulfur-packed bed reactors with excess sulfate rejection by nanofiltration (Asik et al., 2021). Particularly, the membrane biofilm reactor (MBfR) (Nerenberg, 2016; Rittmann, 2018) is promising because it makes efficient delivery of gaseous substrates possible (H_2_ and O_2_ in Eqs. 1 and 4). The MBfR is made up of bundles of hollow-fiber membranes that are hydrophobic and non-porous. A gaseous substrate is supplied to the lumen of the fibers and transferred by diffusion into a biofilm growing on the outer surface of the membrane (Zhou et al., 2019). Table 1 summarizes reported sulfur transformation performances involving gaseous substrates of selected studies focused on sulfur removal. Mining operations require high-rate microbial processes because of the large process-water and effluent flows involved. MBfRs, fluidized bed reactors (FBRs), and gas lift reactors (GLRs) are more commonly used with gaseous substrates (Di Capua et al., 2015; Sinharoy et al., 2020). Because FBRs and GLRs deliver substrate gases by bubbling, they require gas recompression and recycling and have a higher risk of H_2_ and H_2_S off-gassing. The high cost of membranes has been regarded as the main obstacle for MBfR adoption instead (Martin and Nerenberg, 2012), however, the cost of commercial membranes is decreasing and new commercial applications will continue to emerge (Nerenberg, 2016).

**TABLE 1:** Examples of sulfate-reducing and sulfide-oxidizing systems focused on sulfur removal.

Wastewater	Substrate	Reactor type, liquid volume, operating temperature	Average sulfide or sulfur productivities (kg S/m^3^-d) and efficiencies (in parenthesis)	Reference
**Sulfate-reducing reactors**				
Synthetic	80% H_2_/20% CO_2_	GLR/Pumice carrier, 4.5 L, pH 7–7.5, 30°C	4.67–7.07 (77–82%)	van Houten et al. (1994)
				van Houten et al. (1995)
				van Houten et al. (1996)
Cu tailings water	80% H_2_/20% CO_2_	MBfR, 25 ml, pH 7.6, 21°C	0.81 (98%)	Schwarz et al. (2020)
Cu tailings water	95% H_2_/5% CO_2_	MBfR, 25 ml, pH 8.0 ± 0.2, 21 ± 3°C	1.70–3.74 (97–92%)	This study
**Sulfide-oxidizing reactors**				
Synthetic	Air	Expanded bed, 12 L, pH 7.2–7.6, room temp	5.00 (72%)	Janssen et al. (1997)
Sulfidogenic reactor effluent	O_2_	MBfR, 43 ml, pH 7–9, room temp	2.40 (76%)	Sahinkaya et al. (2011)
Synthetic	Air	GLR, 4.9 L, pH 7.6–8, room temp	2.91 (79%)	Lohwacharin and Annachhatre, (2010)
Synthetic	Air	MBfR, 4.1 L, pH 7.5, 30°C	2.0–5.5 (83.7–56.3%)	Jiang et al. (2019)
H2-MBfR effluent	Air/O_2_	MBfR, 25 ml, variable pH and pH 8.0 ± 0.2, 21 ± 3°C	0.70–2.66 (78–48%)	This study

A critical operational aspect of the coupled reducing and oxidizing processes is pH control. A pH that is too low increases the risk of H_2_S toxicity and one that is too high increases the risk of CaCO_3_ scaling (Suárez et al., 2020). To obtain the overall net acid-base demand of the coupled process, the acid-base equivalents in the key reactions must be summed up. Reaction 1 consumes one equivalent of strong acid (H^+^) per mole of sulfate reduced, while reaction 2 also consumes one equivalent of strong acid per mole of S^0^ formed. Hence, under optimal conditions of 100% S^0^ formation, the acid demands of the reducing and oxidizing reactors approach the 1:1 ratio. This is an approximation because in the normal working alkaline pH range some H_2_S in Eqs 1, 2 is unionized and this fraction varies with pH. In addition, reaction 3 shows that further oxidation of S^0^ to SO_4_^2-^, instead of consuming acid, produces 2 equivalents of strong acid per mole of S^0^ oxidized. These insights are critical to the correct design of an automatic pH control strategy, indicating the requirement for acid dosing in the reducing module, and acid and eventually base dosing in the oxidizing module. Also, the preceding acid-base accounting is valid for pH ∼ 8, the chosen operational pH, in which HS^−^ is dominant and H_2_S toxicity is minimized. Other factors and MBfR processes can also affect the pH, such as feed water alkalinity, external CO_2_ gas addition, CaCO_3_ precipitation/dissolution, CO_2_ consumption by autotrophs, and CO_2_ production by heterotrophs (Tang et al., 2011). However, it is expected that these processes will be insignificant given the high metabolic rate for sulfur species and considering that the CO_2_ ratio in the gas mixture is kept low and that the pH of ∼ 8 minimizes the risk of CaCO_3_ scaling. Nevertheless, a pH model should be developed to assess the relevance of these processes under different operating conditions (Xia et al., 2016).

Different pH control strategies have been implemented in MBfRs such as acid injection without automatic control (Schwarz et al., 2020), and CO_2_ dosing as part of the reactive gas mixture (Suárez et al., 2020) or with a separate membrane (Xia et al., 2020). It has been shown that acid/base injection lacking automatic control is prone to overshooting and that CO_2_ dosing has limitations for influents rich in Ca such as mining effluents since the risk of CaCO_3_ scaling increases. Hence, the chosen alternative in this study is acid injection with automatic control for both reducing and oxidizing modules. Although the oxidizing module might demand a base when it is underperforming, this situation can be theoretically avoided by careful O_2_ dosing.

The main purpose of this research was to optimize the operation of coupled reducing and oxidizing MBfR modules for conversion of sulfate-sulfur into elemental sulfur from sulfate-rich mining process water. We assessed the effect of increasing sulfate surface loading, automatic pH control, and hydrogen and oxygen gas pressures, on the removal of sulfate, the amount of S^0^ produced, and pH stability.

## Materials and Methods

### Experimental Design

**FIGURE 1:**
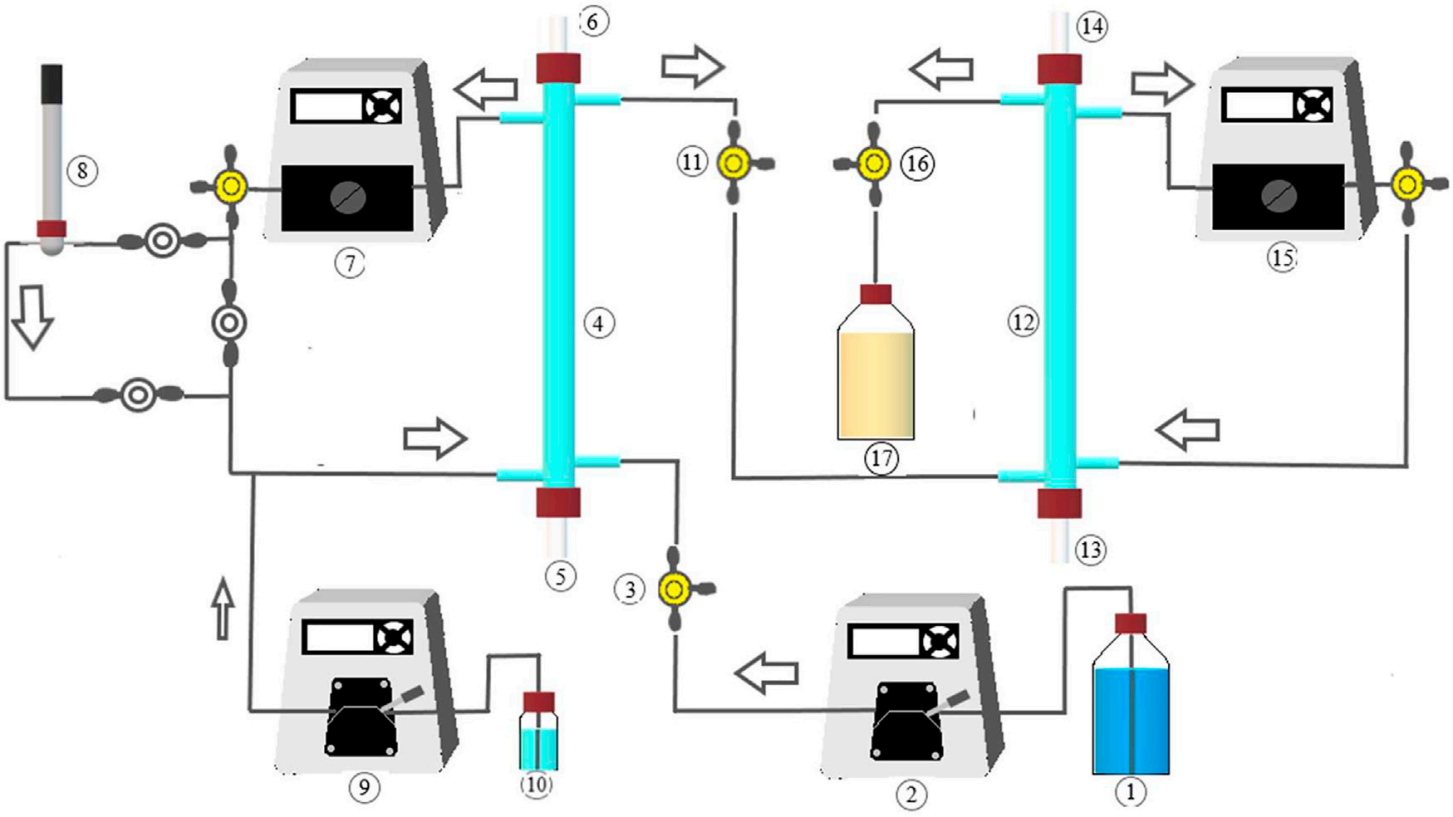
MBfR system implemented for sulfate removal to elemental sulfur. 1: Influent; 2: Influent pump; 3: Influent sampling; 4: H_2_-MBfR; 5: H_2_/CO_2_ feed; 6: H_2_/CO_2_ venting; 7: Recirculation pump; 8: pH electrode and flow-cell; 9: Acid dosing pump (under automatic control); 10: HCl solution; 11: H_2_-MBfR effluent sampling; 12: O_2_-MBfR; 13: Air/oxygen feed; 14: Air/oxygen venting; 15: Recirculation pump; 16: O_2_-MBfR sampling; 17: Effluent.

The reactor system, illustrated in Figure 1, consisted of reducing (H_2_-MBfR) and oxidizing (O_2_-MBfR) MBfR modules connected in series. Both reactor modules were made of 40-cm glass tubes of 9-mm interior diameter, giving an empty bed volume of 25 ml each. The modules were connected with Viton tubing (Masterflex, Cole-Parmer, Vernon Hills, IL, United States), plastic fittings, and three-way valves for influent and effluent sampling. MBfR membranes were selected based on their high gas permeability and current commercial use. The H_2_-MBfR used 17 non-porous polydimethylsiloxane (PDMS) fibers of 460-µm external diameter, resulting in a fiber specific surface area of 393 m^2^/m^3^, while the O_2_-MBfR was made of four strands of 48 fibers each of non-porous polymethylpentene, 80 µm in outer diameter (772 m^2^/m^3^ specific surface area). The experimental set-up included also five peristaltic pumps (Cole Parmer Masterflex L/S pumps and Masterflex L/S Easy-Load II pump heads), one for feeding the influent, two for MBfR-module recirculation mixing, and two for acid solution dosing. The H_2_-MBfR was supplied at one end of the fibers with a mixture of 95% H_2_ + 5% CO_2_ and the O_2_-MBfR was operated with compressed air until day 136 when it was switched to 100% O_2_. The other end of the fibers was kept closed to avoid the loss of H_2_ and O_2_ and the volatilization of H_2_S.

The feed was process water from a copper mine in Central Chile, extracted from the copper sulfide flotation circuit. It is characterized by high equimolar concentrations of sulfate and calcium ( ∼ 16 mM) since the flotation of copper sulfide uses sulfuric acid and lime as reagents. This process water contains traces of other metals (Al, Co., Cu, Fe, and Zn) too. Before being used, it was aerated for wo days, reflecting equilibrium with the atmosphere in a tailing dam, and then bubbled with N_2_ gas to minimize O_2_ entry into the H_2_-MBfR, giving an average feed pH of 6.5. The detailed chemical composition is included in Suárez et al. (2020). To provide nutrients and essential elements, the feed was amended with 23 mg/L of NH_4_Cl, 11 mg/L of KH_2_PO_4_, 1 ml/L micronutrients solution, and 1 ml/L selenite-tungstate solution. The composition per liter of the micronutrients solution was 1.5 g FeCl_2_∙4H_2_O, 70 mg ZnCl_2_, 100 mg MnCl_2_∙4H_2_O, 6 mg H_3_BO_3_, 190 mg CoCl_2_∙6H_2_O, 2 mg CuCl_2_∙2H_2_O, 24 mg NiCl_2_∙6H_2_O, 36 mg Na_2_MoO_4_∙2H_2_O, and 10 ml 25% HCl. The selenite-tungstate solution contained per liter 500 mg NaOH, 4 mg Na_2_WO_4_∙2H_2_O, and 3 mg Na_2_SeO_3_.

The inoculum for the H_2_-MBfR consisted of 12.5 ml of bulk liquid drawn from a previous sulfate-reducing reactor in a solution with 12.5 ml of DSMZ medium 63 (Atlas, 2010), 6.2 g/L of glycerol, and 2.7 g/L of sodium sulfate. The pH was adjusted to 8.1 with 140 µL of 1M NaOH. The H_2_-MBfR was left in recirculation mode with the inoculation solution for 3 days at 1.5 psig (1.1 atm absolute pressure) of gas pressure until sulfide smell in the sampling port was evident. Then, influent feeding began, and the downstream O_2_-MBfR at 3 psig (1.2 atm absolute pressure) air pressure was colonized by H_2_-MBfR effluent. After 10 days of monitoring pH, ORP, and sulfate and sulfide levels, sampling began.

The MBfRs were operated at room temperature (21 ± 3°C), pH 8.0 ± 0.2, and their pH control systems consisted of pH controller (Cole-Parmer, 300 pH/ORP/Temperature), pH flow-through cell (Cole-Parmer, 800 μL, 3 mm ID, glass), pH electrode (Cole-Parmer, combination, double-junction, sealed), a peristaltic pump (Cole Parmer, Masterflex L/S digital drive + Easy-Load II pump head, 100 RPM max), and acid solution (1–6% HCl). The method of adding HCl was based on Schröder-Wolthoorn et al. (2008), who used a 3% HCl solution. The calibration and operation of the pH control system started immediately for the H_2_-MBfR and on day 98 of operation for the O_2_-MBfR.

Table 2 shows the operational conditions during each of the operational phases of the experiment, designed for increasing values of the sulfate surface loading rate (SLR).

**TABLE 2:** Operational phases of the reactors and operating conditions.

Parameter/Operating phase	F1	F2	F3	F4
Days	0–26	27–59	60–108	109–147
SO_4_^2-^ loading (g S/m^2^-d)	4.0	4.5	6.0	10.5
H_2_/CO_2_ gas mixture pressure (psig)	2–10	7–8	6–7	6.5–10
Air/O_2_ gas pressure (psig)^a^	3–12	2.5–13	1.5–4.1	3.0–10

a Pure oxygen was fed to the O_2_-MBfR from day 31 onward.

### Chemical Analysis

Samples were drawn with a 10-ml syringe three times a week from the H_2_-MBfR and O_2_-MBfR effluent sampling ports for measurement of pH, ORP, SO_4_^2-^, and total S^2-^. Immediately, a 5-ml aliquot was used to measure pH and ORP and the remnant was transferred to a capped Eppendorf tube, from where subsamples were extracted for total S^2-^ and SO_4_^2-^ measurement. Periodically, additional effluent samples were extracted for analysis of Ca^+2^ and alkalinity.

The pH was determined with an HQ40d portable meter (HACH, Loveland, CO, United States) and the ORP with an Orion 370 PerpHecT benchtop meter (Thermo Scientific, Waltham, MA, United States), both periodically calibrated. The total S^2-^ concentration was measured using Methylene Blue kits [HACH, based on SM4500-S^2-^ D, APHA (2005)]. Samples were filtered through sterile 0.45-μm pore-size membrane filters (Sartorius, Göttingen, Germany) for measuring SO_4_^2-^ with Sulfaver kits [HACH, based on SM4500-SO_4_^2-^ E, APHA (2005)] and dissolved Ca^2+^ by atomic absorption spectrometry [AAnalyst 400, Perkin Elmer, Waltham, MA, United States SM3500, APHA (2005)]. Alkalinity was determined by sulfuric acid titration with a digital titrator (HACH, method 8203).

### Surface Loading Rates and Fluxes of SO_4_^2−^, S^2−^ and S^0^ Formation

The surface loading rate (SLR) is the loading in weight of SO_4_^2-^ or total S^2-^ that is supplied per unit of membrane area and per unit of time (g S/m^2^-d) to the H_2_-MBfR or O_2_-MBfR, respectively, and can be calculated as follows (Eq. 5):

SLR=Q·CinABF (5)

where Q is the influent flow rate to each module (L/d), C^in^ is the influent concentration of SO_4_^2-^ or total S^2-^ (g S/m^3^) and A_BF_ is the membrane surface area of each module (m^2^).

The respective removal/production fluxes of SO_4_^2-^ or total S^2-^ (J, g S/m^2^-d) were calculated for each module according to Eq. 6:

J=Q·(Cin−Cout)ABF (6)

where C^in^ and C^out^ are the influent and effluent concentrations of SO_4_^2-^ or total S^2-^ (g S/m^3^).

The formation of S^0^ cannot be measured by direct analysis of the effluent as a fraction of the produced S^0^ remains entrapped in the biofilm. Consequently, a mass balance on dissolved sulfur is used to estimate the rate of S^0^ formation ( $J_{S^{0}}$ , g S/m^2^-d) as (Eq. 7):

JS0 = (QABF)⋅[(CS2−in − CS2−out)+(CSO42−in − CSO42−out)] (7)

where A_BF_ is the membrane surface area of the O_2_-MBfR module (m^2^).

The previous expression assumes that the concentrations of intermediates such as sulfite ( $SO_{3}^{2-}$ ) and thiosulfate ( $S_{2}O_{3}^{2-}$ ) are non-detectable, as observed in previous studies (Janssen et al., 1997; Sahinkaya et al., 2011). Under normal operating conditions, $S_{2}O_{3}^{2-}$ does not form during sulfide oxidation. However, under extreme oxygen limitation, when chemical oxidation predominates over biological oxidation, $S_{2}O_{3}^{2-}$ is formed (Janssen et al., 1995). The same is true in halo-alkaline sulfide oxidation, where selectivity for thiosulfate was 3.9–5.5% at pH 8.6 and 20–22% at pH 10 (van den Bosch et al., 2008). Finally, using Eqs 1–4 stoichiometries, the hydrogen ( $J_{H_{2}}$ , g H/m^2^-d) and oxygen ( $J_{O_{2}}$ , g O/m^2^-d) fluxes for the reducing and oxidizing modules, respectively, can be calculated as (Schwarz et al., 2020):

JH2=4⋅JSO42−⋅232 (8)

$$J_{O_{2}}=0.5\cdot J_{S^{0}}-2\cdot J_{SO_{4}^{2-}}$$ (9)

### Biomolecular Analysis

Biofilm samples and effluent from the bioreactor modules were collected at the end of the experiment for the analysis of the microbial community. Membranes connected to each reactor were removed and divided into three parts of equal length to extract genomic DNA as described by Suárez et al. (2020). In addition, suspended biomass from effluents was processed to extract DNA at the end of the experiment. Microbial community profiling was carried out by using the Illumina MiSeq platform (Illumina Inc., San Diego, CA, United States) performed at Genoma Mayor, Santiago, Chile. The 16S rRNA gene analysis targeted the V4 region (forward primer F515- GTGCCAGCMGCCGCGGTAA, reverse primer R806- TAATCTWTGGGVHCATCAGG) using the protocol of Caporaso et al. (2011). For bioinformatics processing of the data, DADA2 v.1.10.0 was performed (Callahan et al., 2016) including quality filtering, error estimation, merging of reads, removal of chimeras, and selection of amplicon sequence variants (ASVs). Taxonomy was assigned in DADA2 to ASV2s using SILVA reference dataset v. 132 (Yilmaz et al., 2014). Sequences were submitted to the National Center for Biotechnology Information (NCBI) Sequence Read Archive (SRA) through Bioproject number PRJNA802795.

## Results and Discussion

### Performance of the H_2_-MBfR

**FIGURE 2:**
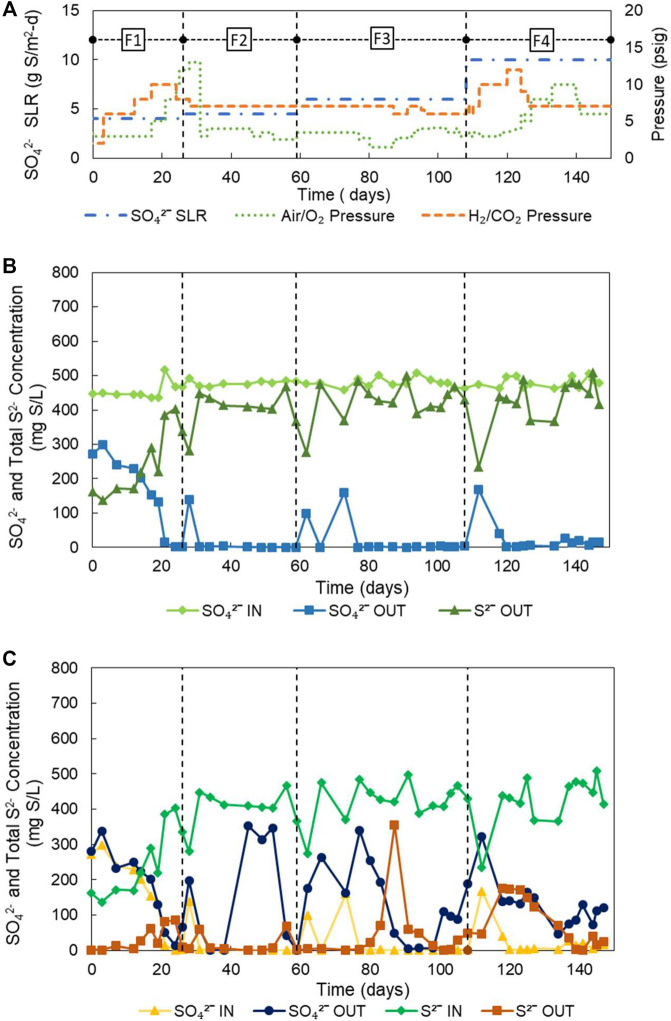
**(A)** Operating conditions and evolution of SO_4_^2−^ and S^2−^ from the H_2_-MBfR and O_2_^−^-MBfR; **(B)** evolution of SO_4_^2−^ and S^2−^ from the reducing stage (H_2_-MBfR) and **(C)** evolution of SO_4_^2−^and S^2-^ from the oxidizing stage (O_2_^−^-MBfR).

As can be seen from the operational conditions of Table 2; Figure 2A, the experimental strategy consisted of gradually increasing the sulfate loading from 4.0 to 10.5 g S/m^2^-d in 4 phases. During each phase optimizations of gaseous pressures were carried out, first adjusting the pressure of the H_2_/CO_2_ mixture for the efficient reduction of sulfate, and then that of air and later O_2_ for the effective transformation of S^2-^ to S^0^. Figure 2A further shows the variation of pressures during the experiment and Table 3 the performances and obtained fluxes.

**TABLE 3:** Performances and fluxes.

Parameter/Operating phase	F1	F2	F3	F4
**Average performances (%)**				
H_2_-MBfR SO_4_^2-^ reduction^a^	62	97	95	92
H_2_-MBfR S^2-^ formation^a^	57	95	95	88
O_2_-MBfR S^2-^ oxidation^b^	88	96	88	78
O_2_-MBfR S^0^ production^c^	89	69	65	62
Overall SO_4_^2-^ removal^a^	53	68	66	65
Overall S^0^ production^a^	47	65	55	46
**Average fluxes (g/m^2^-d)**				
SO_4_^2-^-S	2.33/−0.11^d^	4.32/−0.64^d^	5.74/−0.79^d^	9.54/−1.19^d^
S^2-^-S	−2.27/1.02^d^	−4.23/1.92^d^	−5.73/2.44^d^	−9.19/3.84^d^
S^0^-S^e^	−0.91	−1.28	−1.67	−2.66
H_2_	0.63	1.07	1.44	2.47
O_2_	0.74	2.24	2.81	4.30

a % of influent sulfur.

b % of sulfide formed.

c % of sulfide oxidized.

d Fluxes in H_2_-MBfR and O_2_-MBfR, respectively.

e Specific surface area of the O_2_-MBfR is 96% higher than that of the H_2_-MBfR which explains the relatively low S^0^-S fluxes.

During phase 1, the sulfate load was 4 g S/m^2^-d. At the beginning of the phase, the reactor experienced H_2_ limitation at an H_2_/CO_2_ pressure in the 2-3 psig range. Consequently, only 23% of the sulfate was removed. To increase the sulfate removal rate, starting on day 3, the H_2_/CO_2_ pressure was increased three times up to 10 psig during the first 21 days. The reactor responded to this stepped increase in pressure with the total reduction of sulfate on day 24. Considering that the increase in pressure could have been excessive, at the end of stage 1 and the beginning of stage 2, the H_2_/CO_2_ pressure was gradually decreased to 7 psig, and indeed, no increase in the effluent sulfate concentration was noticed. During phase 1, an average sulfate reduction efficiency of only 62% was obtained, because of the extended H_2_ limiting condition. However, due to the rapid response of the reactor to the increase in pressure of H_2_/CO_2_, before the end of phase 1 (day 24), 100% sulfate removal at a flux of 4.07 g S/m^2^-d was achieved.

In phase 2, the reactor quickly adapted to a 12.5% increase in sulfate loading to 4.5 g S/m^2^-d, not being necessary to increase the H_2_/CO_2_ pressure of 7 psig. Only a slight temporary increase in sulfate concentration was recorded at the beginning of the phase. Then, a steady-state was reached characterized by a stable production of sulfide, reaching a high average removal of sulfate of 97% (4.32 g S/m^2^-d) in the phase. Furthermore, according to Figure 2B, if the initial sulfate peak was eliminated, sulfate removal would have averaged 100%. This stability and efficiency of the H_2_-MBfR can be considered a significant strength of the reactor system.

During phase 3 the sulfate SLR was further increased by 33.3% to 6.0 g S/m^2^-d while keeping the H_2_/CO_2_ pressure constant at 7 psig. The two specific increases in the effluent sulfate concentration at the beginning of this stage, which are again due to the adaptation of the biofilm to the new load, explain the loss of sulfate removal efficiency, which averaged only 95% during phase 3. However, the average sulfate flux increased to 5.74 g S/m^2^-d during this phase. It should be noted that although the combined load increase during phases 2 and 3 was 50%, a permanent increase in H_2_/CO_2_ pressure was not necessary to satisfy the increased demand for H_2_. This shows that in the H_2_-MBfR the delivery of gas by the membrane is demand-driven, which means that gases within certain reasonable pressure ranges are never overdosed (Rittmann, 2018).

Finally, in phase 4 the SLR was increased by 75% to 10.5 g S/m^2^-d, by increasing the inflow, calculated to achieve an HRT of 3 h. Again, after an initial peak of adaptation, the effluent sulfate stabilized at values close to zero. To control the initial peak of effluent sulfate in the face of the significant increase in SLR this time, the H_2_/CO_2_ pressure was temporarily increased to 10 and then to 12 psig. Possibly, the control of the sulfate peak would have been more effective if the H_2_/CO_2_ pressure increase had coincided with the load increase. Once again, the good performance of the H_2_-MBfR was reflected during this stage in the high average sulfate reduction efficiency of 92%, only affected by the initial sulfate peak. During the final phase of the experiment, an average sulfate flux of 9.54 g S/m^2^-d was reached, which represents a very important advance to values ​​of previous MBfR investigations of <2.74 g S/m^2^-d (Ontiveros-Valencia et al., 2012, 2016; Schwarz et al., 2020; Suárez et al., 2020). Likewise, the average H_2_ consumption rate of 2.47 g H_2_/m^2^-d obtained during phase 4 also exceeds the values of the MBfR literature of <0.68 g H_2_/m^2^-d (Zhao et al., 2013a; Zhao et al., 2013b; Schwarz et al., 2020), considering a variety of electron acceptors. This demonstrates the value of using membranes with higher gas permeability in our study.

**FIGURE 3:**
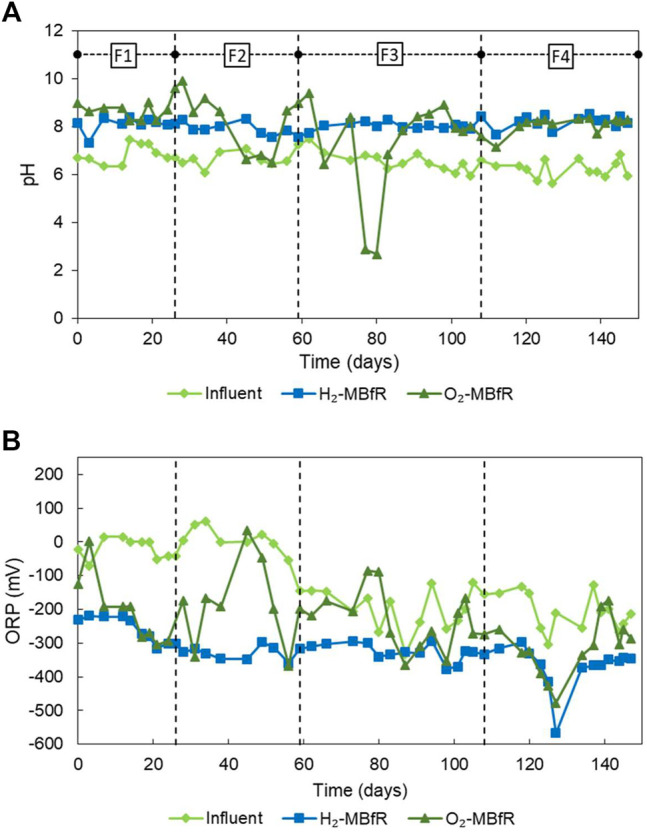
Evolution of **(A)** pH and **(B)** ORP from the H_2_-MBfR and O_2_-MBfR.

Figures 3A,B shows the influent and effluent pH and ORP variations of the MBfRs. In general, good performance of the pH control system is observed in the H_2_-MBfR. In turn, the ORP of the H_2_-MBfR remained practically throughout the experiment below -200 mV, in the appropriate range for SRBs, and most of the time, even in the optimal range of < −270 mV (Hao et al., 2014).

As Table 1 shows, GLRs have achieved the highest volumetric sulfate reduction rates with H_2_, however at steady-state efficiencies of only around 80% in the 7–7.5 pH range, which would not allow complying with the strictest sulfate water-quality standard of 250 mg/L (INN, 1978; European Union, 1998; US EPA, 1999). Nevertheless, van Houten et al. (1995) reached a close to 100% efficiency at pH 8 for a short period, before the reactor failed due to clogging. In this study, the MBfR also achieved high sulfate reduction rates and efficiencies and further performance increases should be tested based on GLR data. Most research on hydrogenotrophic microbial reduction of oxyanions such as sulfate has focused on denitrification (Karanasios et al., 2010; Di Capua et al., 2015). As this study showed, biomass accumulation problems reported in high-rate denitrification MBfRs (Di Capua et al., 2015) will be minimal in the sulfidogenic H_2_-MBfR because with H_2_ the biomass yield of sulfate reduction is about one-third of that of denitrification (Rittmann and McCarty, 2001).

### Performance of the O_2_-MBfR

The operation of a sulfide oxidizing bioreactor is complex because only one control variable, the air or oxygen supply flux, is available to achieve two competing goals, maximizing sulfide oxidation and the formation of S^0^. On the one hand, increasing the oxidation of sulfur requires increasing the supply of oxygen, and on the other, it is necessary to limit the supply of oxygen to favor the formation of S^0^. The efficiency of sulfide oxidation was evaluated by measuring the sulfide concentration and that of S^0^ formation by measuring the sulfate concentration. Sulfide in the effluent was considered as a sign of insufficient oxygen and the formation of sulfate as a sign of excess oxygen. With this criterion, an attempt was made to optimize the operation of the O_2_-MBfR, a task that was complicated by the programmed changes in the H_2_-MBfR operation.

Figure 2C shows the influent and effluent sulfide and sulfate concentrations of the O_2_-MBfR during the experiment. During the first half of phase 1, the effluent sulfide was near zero and the influent and effluent sulfate concentrations were similar, indicating that the 3 psig air pressure was optimal. Then, coinciding with the start of the increase in influent sulfide on day 14, sulfide began to be detected in the effluent, so that during the second half of phase 1 the air pressure was gradually increased from 3 to 12 psig. During phase 1, the reactor performed well, with oxidation efficiencies of S^2-^ of 88% and of production of S^0^ of 89%, which during the first 2 weeks were even higher (97 and 89%, respectively), while the S^2-^ and S^0^ fluxes averaged 1.02 and -0.91 g S/m^2^-d, reaching maximums of 1.54 and -1.47 g S/m^2^-d on day 24.

The O_2_-MBfR performed very well again during the first 2 weeks of phase 2 with S^2-^ oxidation efficiencies of 95% and S^0^ production efficiencies of 91%. As the manufacturer’s recommended maximum operating pressure of 10 psig began to be exceeded, on day 31 the air supply was changed to pure oxygen, generating an increase in oxygen pressure from 2.7 psig (in the air) to four psig. The increase in the effluent sulfate concentration beginning on day 40, indicates an excess of oxygen so that the oxygen pressure was gradually decreased to 2.5 psig between days 46 and 52, achieving the desired sulfate decrease from 343 to 43 mg S/L on day 56 but with an increase in sulfide from 7 to 69 mg S/L. The sulfate and sulfide concentrations decreased to 0 and 3 mg S/L, respectively, on day 59 due to a decrease in influent sulfide. Therefore, small variations in the concentration of the influent sulfide can alter the redox balance, improving or deteriorating the performance of the reactor, requiring continuous adaptation of the oxygen pressures to the variations in the sulfide load.

In phase 2, due to the excessive supply of oxygen, the average efficiency of sulfide oxidation improved slightly compared to the previous stage (96%) while the average efficiency of S^0^ production significantly worsened (69%). Due to the increase in the sulfide load, however, the average S^2-^ and S^0^ fluxes increased to 1.92 and −1.28 g S/m^2^-d, respectively.

Phase 3 was characterized by a significant increase in the sulfide load, for which an increase in oxygen pressure from 2.5 to 3.5 psig was carried out at the beginning of the phase (day 60). Initially, as the H_2_-MBfR was slow to respond to the increase in sulfate loading with a higher production of sulfide, the increase in oxygen pressure generated an over-oxygenation condition in the O_2_-MBfR. As a result, the effluent sulfate increased significantly, which could be controlled by reducing the O_2_ pressure to 1.5 psig between days 78 and 80. At this oxygen pressure, the oxidizing capacity was insufficient, generating a rapid increase in the effluent sulfide, and therefore the pressure was gradually increased to four psig from day 87 to day 98 when a minimum of sulfate and sulfide effluent of 7 and 13 mg S/L was achieved. However, towards the end of phase 3, there was a slight increase in the effluent sulfate that was not eliminated before the start of phase 4. Because with each adjustment of the oxygen pressure carried out, either too oxidizing or too reducing conditions were generated during phase 3, without achieving a stable optimum point, the oxidation efficiencies of S^2-^ and formation of S^0^ reached values of only 88 and 65%, respectively, although the corresponding average fluxes continued to increase (2.44 and 1.65 g S/m^2^-d).

Finally, in phase 4, faced with the increase in the influent load of sulfate to the H_2_-MBfR, an operating strategy different from that of the previous phase was tried, leaving the O_2_ pressure constant during the first 2 weeks, pending that the H_2_-MBfR responded to the increase in sulfate loading with an equivalent increase in sulfide flux. Only on day 118, when a high level of sulfide was detected in the O_2_-MBfR effluent, the O_2_ pressure began to be increased from 3 psig to five psig on day 127, but without achieving a decrease in the effluent S^2-^. Therefore, the O_2_ pressure was successively increased to 8 psig on day 133, and then to 10 psig on day 137, until the sulfide disappeared on day 139. During this phase, average efficiencies of oxidation of S^2-^ of only 78% and production of S^0^ of 62% were achieved, although they were obtained at maximum average fluxes of S^2-^ of 3.84 g S/m^2^-d and S^0^ of −2.66 g S/m^2^-d. There is the potential to increase these fluxes significantly, as Sahinkaya et al. (2011) reported S^0^ fluxes of −9.6 to −48 g S/m^2^-d at O_2_ pressures of 3–15 psig using MHF 200 TL membranes (Mitsubishi Rayon).

During the first 3 phases, the O_2_-MBfR did not have a pH control system, so it varied freely. The production of S^0^ consumes 1 mol of weak acid for each mole of S^0^ produced, so the pH tends to rise when the efficiency of S^0^ production is high (Eq. 2) and to decrease when sulfate production dominates (Eq. 3), as shown in Figure 3A. It was considered that S^2-^ loading increases could cause pH increases and generate fouling of the membrane due to precipitation of CaCO_3_ on the membrane, so on day 98, an automatic pH control system was installed. In Figure 3A it can be seen that the pH of the O_2_-MBfR became more stable after installation. According to Lin et al. (2018), ORP levels from −400 to −137 mV are optimal for the formation of S^0^. Indeed, when ORP levels exceeded the −137 mV limit twice, on days 45–49 (34 mV > ORP > -46mV) and days 77–80 (−86 mV > ORP > −89 mV), the worst yields of S^0^ formation were obtained. These two events were further characterized by high sulfate concentrations and low pH levels. However, these two very time-limited events do not explain the observed levels of S^0^ formation inefficiency, so it remains to be determined, which are the optimal levels of ORP in O_2_-MBfR reactors for S^0^ formation.

### Overall Performance of the MBfR System

The best average global transformation yield of influent sulfate into elemental sulfur was 65%, achieved during phase 2. This phase was characterized by a sulfate reduction process that was stable and also had the highest average yield (97%). This stability of the H_2_-MBfR could also contribute to the oxidation efficiency of the O_2_-MBfR being the highest (96%). As mentioned before, the exchange of air for oxygen affected the efficiency of S^0^ formation (69%), explaining the overall performance of phase 2. Specifically, based on data from days 34 and 38 of phase 2, the average global transformation yield reached 98%. During phase 3, on days 91–105, a good overall performance of 85% transformation from SO_4_^2-^-S to S^0^ was achieved. Also, during stage 4, on days 134–147, the overall transformation yield from SO_4_^2-^-S to S_0_ was 74%. Based on these good punctual results, future studies should evaluate automatic oxygen pressure control strategies in the O_2_-MBfR, based on the ORP for example, because the process of S^0^ formation is very sensitive to this variable. Also, the recovery of S^0^ particles from the O_2_-MBfR must be still systematically evaluated. As the MBfR modeling study of Jiang et al. (2019) showed, S^0^ can make up to 80% of the biofilm dry weight if it is assumed that S^0^ remains attached to the cells once excreted.

### Bacterial Community Diversity

**FIGURE 4:**
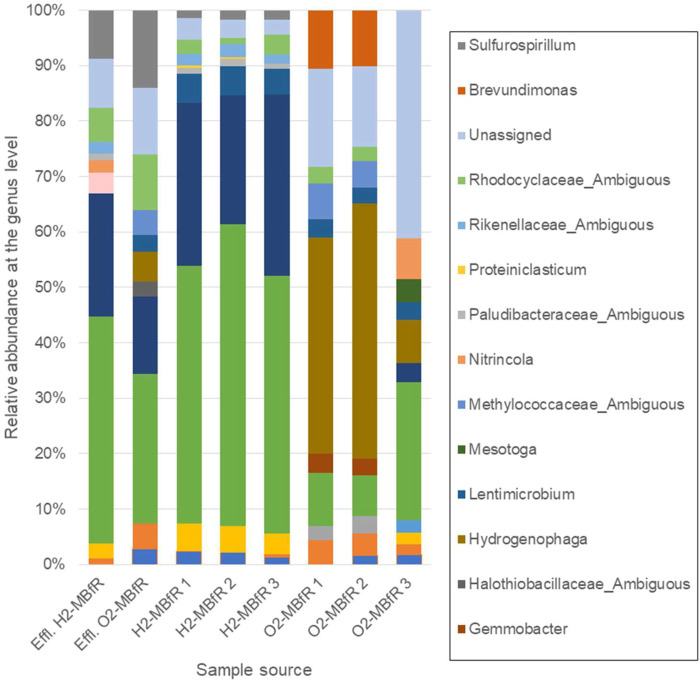
Relative abundances at the genus level for the reducing stage of the sequential system (H_2_-MBfR), obtained from an effluent sample and three sections of fiber and for the oxidizing stage (O_2_-MBfR), obtained from an effluent sample and three sections of fiber taken at the end of the operation. Fiber section order in both cases is 1, 2, and 3 from bottom to top, Introduction being closest to the gas feed.

The majority of the sequences in the anaerobic module were affiliated mainly to two genera, *Desulfomicrobium* and *Desulfovibrio*, both from the order *Desulfovibrionales* (Kuever, 2014), which members are known as sulfate-reducers. Their relative abundance was 77.6 ± 1.6% at the end of the operation, where sulfate removal was completely achieved (Figure 4). The effluent collected in the H_2_-based MBfR showed that the dominant phylotypes were from the same SRB genera, although their abundance decreased to 63.2% while those of the *Sulfurospirillum* genus increased the most from 1.6 ± 0.2–8.8%. This genus consists of versatile, often microaerophilic bacteria, growing with many different substrates where electron donors can be hydrogen, sulfide, and organic acids, while electron acceptors under anaerobic respiration are nitrate, fumarate, and sulfur species other than sulfate (e.g., thiosulfate, elemental sulfur, polysulfides) (Goris and Diekert, 2016). Dissolved oxygen in the influent or diffused through the flexible tubing could perhaps result in sulfur partial oxidation products which were then reduced to sulfide by *Sulfurospirillum* (Lin et al., 2018). This overrepresentation of the *Sulfurospirillum* genus in the effluent compared to the fibers could then be due to flexible tubing biofilms. Carbon dioxide utilization is not a physiological feature found in *Sulfurospirillum* spp., though its growth can be promoted by the soluble microbial products (SMP) released by the SRB (Ontiveros-Valencia et al., 2018; Schwarz et al., 2020). Besides, Sánchez-Andrea et al. (2020) demonstrated recently that *Desulfovibrio* ssp. secrete formate and acetate while growing autotrophically with H_2_ and sulfate. Hence, *Sulfurospirillum* could have also grown anoxically with both formate and H_2_ as electron donors; acetate as the carbon source; and influent nitrate (0.13 mM) in the H_2_-MBfR, and S^0^ in the O_2_-MBfR, as electron acceptors (Stolz et al., 2015). Finally, in a high-rate S^0^ reducing system to treat sulfate-rich metal-laden wastewater (Sun et al., 2018), the predominant sulfidogenic bacterium was also *Desulfomicrobium*. With just S^0^, *Sufurospirillum* was initially dominant but as high-rate sulfate feeding (at levels comparable to our study) began, *Desulfomicrobium* replaced *Sulfurospirillum*.

In the sulfide oxidation stage, the bacterial population composition showed significant changes, particularly in Introduction, Materials and Methods exposed to the higher pressure of O_2_, where the most represented phylotypes were related to *Hydrogenophaga*, with a relative abundance of 38 and 46% respectively (42.1 ± 5.4% overall). Described members of this genus are chemo-organotrophic or chemolithoautotrophic, growing by the oxidation of H_2_ with CO_2_ as a carbon source (Willems and Gillis, 2015); therefore, its growth in the sulfide oxidation stage may be due to carrying over of H_2_/CO_2_ to the oxidizing biofilm. However, this genus may also have a key role in oxidizing reduced sulfur species. *Hydrogenophaga* spp. have been identified as members of H_2_S oxidizing communities in wastewater treatment (Cytryn et al., 2005; Vannini et al., 2008; Li et al., 2020) and described as a dominant SOB in a full-scale H_2_S-bioscrubber (7.45% relative abundance) (Haosagul et al., 2020). Recently, colorless SOBs were characterized in environmental samples using functional marker genes (Luo et al., 2018; Jaffer et al., 2019). Several sulfide oxidation pathways for conserving energy and S^0^ formation have been described, including the sulfur oxidation (Sox) and dissimilatory sulfite reductase (Dsr) systems (Madigan et al., 2019), and sulfide quinone oxidoreductase (SQR) and flavocytochrome c (Fcc) systems (Dahl, 2020). These systems or their elements are universally distributed among sulfur chemolithotrophs due to lateral gene transfer and pathways can even be redundant (Dahl, 2020). Jaffer et al. (2019) showed that most of the *soxB* gene clone sequences were affiliated to the genus *Hydrogenophaga*, while Luo et al. (2018) found that *soxB* and *sqr* genes were also predominantly expressed in *Hydrogenophaga*.

On the other hand, the genus *Brevundimonas*, capable to grow by using organic carbon released by autotrophic bacteria and determined previously in an anoxic biotrickling filter for H_2_S removal (Khanongnuch et al., 2019) and an airlift bioreactor for biogas desulfurization (4.38% relative abundance) (Quijano et al., 2018) was second in abundance in the sulfide oxidation stage (10.3 ± 0.2% considering Introduction, Materials and Methods). Interestingly, in Results and Discussion and more pronounced in the effluent of the oxidizing module, SRB phylotypes belonging to the genera *Desulfomicrobium* and *Desulfovibrio* carried over from the reducing stage were dominant (28.4 and 41.0% abundance, respectively) which may be closely associated with the depletion of oxygen, promoting reducing conditions. On the contrary, *Hydrogenophaga* phylotypes reached only 7.7% in Results and Discussion of the oxidizing module and 5.4% in the effluent.

### Treatment Economics and Sustainability

The electron donor cost makes up about one-half of the operational cost of sulfate reduction, giving costs of 0.26 and 0.20 US$/kg SO_4_ for ethanol and H_2_, respectively (Bijmans et al., 2011). Besides being cheaper, as solar or wind-based production systems become widespread, green H_2_ will be also more sustainable (Acar and Dincer, 2018). Green H_2_ is one of the key fuels that will help tackle the critical energy challenges of a wide range of sectors including long-haul transport, chemicals, iron and steel, and storage of electricity from renewables (IEA, 2019). Consequently, countries worldwide are investing heavily in the research and development of green hydrogen-based technologies for emissions reduction (UNEP, 2020). Armijo and Philibert (2020) estimate a very competitive short-term cost of green H_2_ for Chile of around 2 US$/kg H_2_ (both solar and wind-based), in the range of 1.60–2.05 US$/kg H_2_ reported for coal-based production with carbon capture and storage in Canada (Yu et al., 2021). Considering that 4 mol of H_2_ are required to reduce each mole of sulfate, a green H_2_ cost of 0.17 US$/kg SO_4_ is obtained (equal to 0.27 US$/m^3^ if the sulfate content of the tailings water of 1.5 g/L is considered). Since H_2_ is the main operating cost item of reducing MBfRs, this value compares favorably with the cost for technologies such as reverse osmosis of 0.5–2.5 US$/m^3^ (World Bank, 2019) which in addition generates a residual stream. It must also be considered that for the medium and long term a significant reduction in the cost of H_2_ is expected because of technological development and its mass production (IRENA, 2020). The production cost of green hydrogen depends mainly on the cost of the renewable power, the intermittency of its supply, and the cost of the electrolyser (CEFC, 2021). Furthermore, transport and storage requirements may have to be factored in the cost of delivered hydrogen. The electrolyser CAPEX is also dependent on the installed capacity, varying from 450 to 850 US$/kW for input energies of 100 and 2 MW in the case of alkaline electrolysers, respectively (Proost, 2019). We estimate that a 100 L/s desulfurization plant for a large tailing facility would demand an energy input for onsite H_2_ production of 2 MW, having then an electrolyser CAPEX of 850 US$/kW, while the above reported cost of green H_2_ for Chile assumes an electrolyser CAPEX of 600 US$/kW. For smaller electrolysers the CAPEX in US$/kW varies even more with scale (Proost, 2019).

The cost of HCl is not negligible but amounts only to 0.027 US$/kg SO_4_, assuming a bulk price of 35 US$/ton HCl. H_2_ produced by water hydrolysis has also the added benefit of the O_2_ produced, valued at 0.24 US$/kg H_2_ in Chile (Armijo and Philibert, 2020). Because hydrolysis generates 0.5 mol of O_2_/mol H_2_, and the proposed system requires 0.125 mol of O_2_/mol H_2_ (reactions 1 and 2), the sale of the 75% surplus O_2_ could generate savings of 0.18 US$/kg H_2_. Besides, the generated S^0^ can be recovered and sold as fertilizer (fourth-quarter 2020 price of 69 US$/ton; U.S. Geological Survey, 2021) or used in bioleaching of ore, contributing to the sustainability of the treatment.

## Conclusion

A coupled hydrogenotrophic-aerobic MBfR system was optimized for sulfate removal and elemental sulfur production in mining-impacted water. By implementing automatic pH control the H_2_-MBfR achieved high average volumetric sulfate removals of 1.7–3.74 g S/m^3^-d at 92–97% efficiencies, close to performances reported for high-rate gas lift reactors but avoiding gas recycling and recompression and minimizing H_2_ off-gassing risks. In addition, biomass accumulation was not a problem and H_2_ supply was on-demand further simplifying gas management. The O_2_-MBfR achieved lower average S^0^ formation performances of 0.7–2.66 g S/m^3^-d at 48–78% efficiencies instead, when compared to expanded bed or gas lift reactors. Both MBfRs can be further optimized, particularly the O_2_-MBfR, by automatizing the feed control and studying biomass and S^0^ accumulation control measures. Finally, an economic evaluation shows that the coupled MBfR technology is cost-effective and that it can be also more sustainable based on current green hydrogen and oxygen price projections.

## Data Availability

The original contributions presented in the study are included in the article, further inquiries can be directed to the corresponding author.
